# Knowledge of actions of inhaled corticosteroids in patients who did not persist drug treatment early

**DOI:** 10.1007/s11096-012-9611-9

**Published:** 2012-01-31

**Authors:** Tanja T. Menckeberg, Jacqueline G. Hugtenburg, Jan Willem Lammers, Jan A. M. Raaijmakers, Marcel L. Bouvy

**Affiliations:** 1 Division of Pharmacoepidemiology and Clinical Pharmacology, Department of Pharmaceutical Sciences, Utrecht University, Utrecht, The Netherlands; 2 Department of Clinical Pharmacology and Pharmacy, VU University Medical Center, De Boelelaan 1117, 1081 HV Amsterdam, The Netherlands; 3 Department of Pulmonary Diseases, Utrecht Medical Centre, Utrecht University, Utrecht, The Netherlands; 4 SIR Institute for Pharmacy Practice and Policy, Leiden, The Netherlands

**Keywords:** Asthma, Community pharmacy, Discontinuing treatment, Disease control, Inhaled corticosteroids, Knowledge of drugs, Netherlands

## Abstract

*Objective* To evaluate, among new users of inhaled corticosteroids that did not persist treatment, knowledge of inhaled corticosteroids' actions and whether they were instructed on the use of their inhaler. *Setting* Fifteen community pharmacies in The Netherlands. *Methods* Patients were interviewed by telephone. Their general practitioners provided diagnostic information and automated dispensing records were retrieved. *Main outcome measures* Knowledge of patients about the actions of inhaled corticosteroids. *Results* 230 (80.1%) of 287 patients were willing to participate. The majority (79.1%) of 230 patients was not aware of the anti-inflammatory actions of inhaled corticosteroids. Most patients were instructed on the use of their inhaler, predominantly by their physician (53%) or pharmacy (35.2%). *Conclusions* Although most patients reported inhaler instruction by at least one health care provider, the majority was unaware of inhaled corticosteroids' actions. Physicians and pharmacists should reconsider the instructions they provide especially to patients who should continuously use inhaled corticosteroids.

## Impact of findings on practice

- The majority of new users of inhaled corticosteroids (ICS) who did not persist treatment were unaware of ICS’ anti inflammatory actions.
- Physicians and pharmacists should reconsider the instructions they provide to patients who should continuously use ICS.

## Introduction

Educating patients on the self-management and thus on actions and correct use of their medication is a fundamental component of asthma management guidelines [1]. Studies on continuous use of inhaled corticosteroids (ICS), showed low persistence and adherence rates varying from 17 to 60% [2–5]. Persistence is generally even lower among new users [6–8]. It has also been shown that patients who do not regularly use ICS, have poor asthma control [9, 10].

In general, non-adherence is associated with a lack of patients’ knowledge about the disease and treatment [7, 11–13]. Studies among patients starting new medication for chronic conditions show that most patients frequently experience practical difficulties with taking their medication [7, 14]. However, these studies did not include patients with respiratory conditions or those who were prescribed ICS.

The high incidence of early discontinuation of ICS in new users could be related to inadequate instructions of patients about the anti-inflammatory properties of ICS and the potential preventive effect of chronic ICS treatment on the occurrence and severity of exacerbations.

## Aim of the study

The aim of the present study was to evaluate the knowledge of ICS actions, among new users of ICS that discontinued treatment. Additionally we aimed to identify which factors influenced knowledge about ICS.

## Method

### Study design

A cross-sectional study in community pharmacies in The Netherlands. Fifteen pharmacies from three clusters of pharmacies both in highly urban areas, in urban and rural areas were included.

### Participants

New ICS users were defined as patients who did not fill an ICS prescription in the 2 years before a first ICS prescription. Early discontinuation was defined as the absence of an ICS refill within at least 6 months after this first prescription. In The Netherlands patients are allowed to be dispensed a supply of canisters sufficient for a maximum period of 3 months. Consequently, we determined discontinuing of ICS after a grace period of 3 months, a total period of 6 months. The majority of patients in The Netherlands visit the same community pharmacy, independently of prescriber. Pharmacy records are therefore virtually complete with regard to prescription drugs [15].

### Procedure

Patients were contacted by telephone by their pharmacist. For patients that could not be contacted during pharmacy opening hours, at least one new attempt was made in evening hours. The telephone interview was conducted using a structured questionnaire. For patients aged less than 14 years, the patients’ caregiver was interviewed.

The general practitioner (GP) of eligible patients were asked to provide information on the diagnosis as well as on the severity of symptoms using a questionnaire. To reduce GPs workload, each GP was asked to provide the information of a maximum of randomly selected 20 patients.

### Privacy

The research was conducted in accordance with the requirements of the Institutional Review Board (IRB). According to the IRB informed consent was not required. The questionnaires were anonymised by use of a randomly assigned unique number for each patient. During the interview, patients were specifically asked for their consent to use the data anonymously for research purposes.

### Questionnaires

#### Questionnaire for patient interview

One questionnaire referred to knowledge of the inhalers’ action and on the health care providers that actually instructed the patient on the use of the inhaler. Questions were asked by means of open-ended questions. The answers were coded by the researchers. In this respect we were relatively forgiving e.g. when patients mentioned an ICS was a preventer we coded this as proper knowledge although they did not specifically call it a corticosteroid or antiinflammatory drug. In addition, a 6-item version of the Asthma Control Questionnaire (ACQ; without FEV_1_) was used to assess disease control. An ACQ score of 1.5 or higher was regarded as possibly not well-controlled disease [16].

### GP questionnaire

The questionnaire for the GPs comprised questions on diagnosis, disease severity and whether patients should have continued ICS use.

### Statistical analysis

Descriptive statistics were calculated for selected patients. Conditional logistic regression was applied to analyse the association between adequate ICS knowledge and potentially confounding variables. *P* < 0.05 was considered statistically significant.

## Results

### Baseline characteristics of responders

Within the 15 participating community pharmacies 287 (42.4%) out of 677 new ICS users did not refill any ICS prescription within 6 months after the first ICS prescription. Out of these 287 eligible patients, pharmacists interviewed 230 (80.1%).

There were no statistically significant differences in age, gender and medication use between 230 patients interviewed and 57 patients not participating.

About half the sample (49.1%) was older than 45 years. The majority of the patients (65.7%) received at least one prescription for any bronchodilator in the year preceding the survey.

### Inhalation instruction

The majority of the patients (53%) recalled to have been instructed either by their prescriber or by their pharmacist (35.2%) (Table 1). 35.2 of patients from each pharmacy claimed to have been instructed by their pharmacist. The percentage varied from 0 to 91% between pharmacies.

**Table 1:** Demographic characteristics, questionnaire items and medication use for the total population

	Total 230 patients (100%)
Gender (% female)	139 (60.4%)
Average age(±SD)	46.3 (±25.5)
ACQ-score^a^	
<1.5 (probably well controlled)	186 (80.9%)
≥1.5 (probably not well controlled)	30 (13.0%)
Drug effects ascribed to ICS	
Patient could not recall a clear mode of action	102 (44.3%)
Bronchodilatation	66 (28.7%)
Anti-inflammatory	33 (14.3%)
Bronchodilatation and anti-inflammatory	15 (6.5%)
Antitussive or mucolytic effect	14 (6.1%)
Inhalation instruction	
Patient could not recall instruction	41 (17.8%)
Physician (GP or pulmonologist)^b^	122 (53.0%)
Pharmacist^b^	81 (35.2%)
GP assistant/nurse^b^	10 (4.3%)
Only information leaflet	11 (4.8%)

^a^Excluding 14 patients with ≥1 missing item in the ACQ

^b^35 patients (15.2%) received instruction by more than one healthcare provider

### Knowledge of ICS

A substantial part of patients (44.3%) was unable to mention the effects of ICS. A minority (14.3%) stated that the effect of ICS was anti-inflammatory. Fifteen patients (6.5%) attributed both bronchodilating and anti-inflammatory effects to ICS (6 of these patients used an inhaler containing a combination of both an ICS and bronchodilator) (Table 1). Patients may perceive that an anti-inflammatory effect will also lead to bronchodilation. Therefore, these patients were also considered to have adequate knowledge. In total, 79.1% patients were giving incorrect answers.

Patients who were aware of the anti-inflammatory actions of ICS were younger (OR 0.98 [0.96–0.99]) and tended to be more often female (OR 1.6 [0.9–3.6]) (Table 2). There was no association between patients’ self reported symptoms (measured by the ACQ) or concomitant use of bronchodilators and knowledge of ICS’ actions. Self reported instruction either by physician, pharmacy or both did not seem to affect patients knowledge of ICS’ actions (Table 2).

**Table 2:** Comparison of patients who were aware of anti-inflammatory actions of ICS and those who were not aware of anti-inflammatory actions of ICS

	Knowledge of ICS’ actions	No knowledge of ICS’ actions	Crude OR OR (95% CI)	Adjusted OR OR (95% CI)
Sample of patients interviewed by telephone (n = 230)	n = 48	n = 182		
Gender (% female)	33 (68.8%)	106 (58.2%)	1.6 (0.8–3.1)	1.9 (0.9–3.6)^a^
Age, years mean ± SD	34.0 ± 19.7	44.7 ± 24.5	0.98 (0.97–0.99)	0.98 (0.96–0.99)^a^
ACQ score, mean ± SD	0.67 ± 1.5	0.45 ± 1.0	1.3 (0.9–1.7)	1.2 (0.9–1.7)^a^
Asthma according to patient	5 (10.4%)	17 (9.8%)	1.1 (0.4–3.0)	–
Instruction				
Physician	21 (43.8%)	101 (55.5%)	0.6 (0.3-1.2)	–
Pharmacist	20 (41.7%)	61 (33.5%)	1.4 (0.7–2.7)	1.2 (0.6–2.4)^a^
No verbal instruction	11 (22.9%)	40 (22.0%)	0.9 (0.4–2.0)	–
Sample of patients of whom the GP was interviewed (n = 115*)	n = 27	n = 88		
Gender (% female)	19 (70.4%)	49 (55.7%)	1.9 (0.7–4.8)	2.7 (0.7–7.8)^b^
Age, years mean ± SD	34.1 ± 21.5	46.3 ± 26.3	0.98 (0.96–1.0)	0.97 (0.94–0.99)^b^
Diagnosis or suspicion of asthma	16 (59.3%)	51 (58.0%)	1.1 (0.4–2.5)	0.7 (0.2–2.3)^b^
ICS intended for chronic use	6 (22.2%)	22 (25.0%)	0.9 (0.3–2.4)	1.3 (0.4–4.0)^b^

* Twenty-one of 40 GPs were willing to participate in the study. In addition, each GP was asked to provide the information of a maximum of 20 patients. Therefore, the GP information was only available for 115 patients

^a^adjusted for all variables with more than 5 events per variable; replacing ‘pharmacist instruction’ by ‘GP instruction’ or ‘no instruction’ did not change the model appreciably

^b^Additional adjustment for Asthma diagnosis and ICS intended as chronic medication

### Asthma diagnosis and disease severity according to GP

Twenty-one of 40 GPs were willing to participate in the study. Consequently, questionnaires on diagnosis and symptom severity of 115 of the 230 participating patients were received. Physicians suspected 67 (58.3%) patients of having asthma. Twelve (11.2%) of these patients had not well-controlled asthma. According to the GPs, 28 patients should not have discontinued ICS treatment.

Of these 115 patients, 88 (76.5%) were not aware of the anti-inflammatory effects of ICS. There was no association between suspicion of asthma by the GP and knowledge of ICS. Except age, none of the other determinants studied, was significantly associated with unawareness of ICS actions (Table 2).

## Discussion

This study shows that the majority of patients who early discontinued the use of ICS lacked knowledge about the potential anti-inflammatory effects of ICS. Patients who were aware of the anti-inflammatory actions of ICS were younger and tended to be more frequently female. Age and gender differences in asthma knowledge have been reported previously [17, 18].

Knowledge of the anti-inflammatory actions of ICS was not influenced by either an asthma diagnosis or the experience of symptoms measured by the ACQ.

Recall bias may be a limitation of the study, as at least 6 months elapsed between the telephone interview and the index ICS prescription. However, most patients did not opt that they could not recollect the answer to our questions. Patients may be aware of ICS actions, but not able to put them into words by themselves, and they may be influenced by response categories. Hence the conclusions about patient awareness will depend on the interview method.

As the study does not compare with patients who continue their treatment, the study has no possibility of determining to what extent lack of knowledge on ICS actions can explain discontinuation. You might find the same lack of knowledge in persistent patients, which would lead to other interpretations of the role of knowledge about ICS actions in ICS persistence. Selection bias might also have occurred as the response rate among GP’s was about 50%.

Nevertheless, the large proportion of patients with low ACQ scores and without high use of bronchodilators suggests that the majority of the patients might have discontinued ICS appropriately. The initial indication for the use of ICS might be partly off-label, such as cough. It is therefore possible that, for these patients, the physician did not consider it necessary to explain ICS’ actions. However in the subgroup of patients with a confirmed GP asthma diagnosis, knowledge of ICS actions did not differ from patients without an asthma diagnosis. Even patients of whom the GP indicated that they should have continued using ICS did not have more knowledge on ICS actions.

Apparently, instructions by health care providers are mainly focused on inhalation technique, as almost all patients claimed to be instructed by at least one health care provider. Patients most frequently mentioned the physician as their instructor of inhaler technique. One third of patients stated that they were instructed by the pharmacy on the use of the inhaler, somewhat higher than reported by Mehuys and co-workers [19]. Nevertheless, there is significant opportunity to increase pharmacists’ instructions of patients. This study showed that being instructed was not associated with increased asthma knowledge. It is important to move instruction beyond inhalation technique and also address the purpose and importance of regular ICS use. Information given to patients can be reinforced by different health care providers [20]. As this study shows that a considerable number of patients have less clear indications for the use of ICS, physicians and pharmacists need to cooperate to identify those patients that are most likely to benefit from monitoring and instructing.

## Conclusion

Although most patients reported inhaler instruction by at least one health care provider, the majority was unaware of inhaled corticosteroids’ actions. Physicians and pharmacists should reconsider the instructions they provide especially to patients who should continuously use inhaled corticosteroids.
